# Hidden Communication Needs in Higher Education: A Scoping Review of Developmental Communication Disorders, Mental Health, and Academic Participation

**DOI:** 10.3390/healthcare14121790

**Published:** 2026-06-21

**Authors:** Xiaowen Qi, Yang Zhao

**Affiliations:** 1 College of Foreign Languages, University of Shanghai for Science and Technology, Shanghai 200093, China; qixiaowen@usst.edu.cn; 2 School of English Studies, Shanghai International Studies University, Shanghai 200083, China

**Keywords:** developmental language disorder, stuttering, university students, hidden disability, inclusive assessment, disability support, academic belonging

## Abstract

Background/Objectives: Higher education requires students to communicate in complex academic and social contexts, including oral presentations, group work, help-seeking, assessment, and peer interaction. For students with developmental communication disorders, and communication-related developmental profiles, these demands may create hidden participation vulnerabilities that affect mental health, academic engagement, and belonging. This scoping review mapped empirical evidence among tertiary students, focusing on mental health, academic participation, social belonging, institutional support, and contextual influences. Methods: A scoping review was conducted in accordance with PRISMA-ScR guidance. Five databases, PubMed, PsycINFO, CINAHL, Scopus, and Web of Science, were searched for English-language, peer-reviewed empirical studies published from 2000 onwards. Eligible studies involved university, college, or tertiary students with developmental speech, language, fluency, pragmatic communication, or communication-related developmental profiles, who reported at least one mental health, academic, or social participation outcome. Data were charted and synthesised thematically, with methodological quality appraised using CASP-informed criteria. Results: Twenty-one studies were included. Evidence was strongest for stuttering and fluency-related participation, while research on developmental language disorder, speech sound disorder, pragmatic language impairment, cluttering, and mixed communication profiles was limited. Across studies, communication needs intersected with anxiety, depression, stress, self-efficacy, oral assessment, help-seeking, disclosure, stigma, accommodation access, and belonging. Key limitations included reliance on self-report, cross-sectional or retrospective designs, inconsistent diagnostic confirmation, and limited evidence for intervention. Conclusions: The available evidence suggests that developmental communication disorders and communication-related developmental profiles can function as hidden participation vulnerabilities in higher education. These vulnerabilities are shaped by students’ communication profiles and by communication-intensive university environments. Universities may therefore need communication-accessible teaching, flexible assessment, visible support pathways, and coordinated support across disability services, counselling, academic support, and speech–language pathology.

## 1. Introduction

Higher education is often framed around access, achievement, and retention, but it is also a communication-intensive environment. Students are expected to process complex spoken and written information, contribute to discussions, complete oral presentations, negotiate group work, seek help, interact with staff, and participate in the social life of the university. These demands shape not only academic performance but also confidence, belonging, disclosure, and access to support. For students with developmental communication disorders, such expectations may create difficulties that are not readily visible to peers, lecturers, disability services, or mental health professionals.

Developmental communication disorders are persistent difficulties affecting language, speech, fluency, and pragmatic communication that originate during development and may continue into adolescence and adulthood. Developmental language disorder (DLD), for example, involves language difficulties that are likely to persist and have functional impact [1]. Stuttering, cluttering, speech sound disorder, and social communication difficulties may also continue to affect educational participation beyond childhood, particularly in academic contexts where competence is often demonstrated through fluent, rapid, confident, and socially flexible communication. Most research and service discussions have focused on childhood and school years, meaning that the implications of these conditions in higher education remain less visible.

The mental health relevance of developmental communication disorders is increasingly evident. Adults with DLD have described mental health difficulties shaped by masking, fatigue, social disconnection, adverse educational experiences, and limited understanding from education, health, and employment systems [2]. Recent reviews have also linked language and communication disorders with social–emotional difficulties and quality-of-life concerns, particularly across childhood and adolescence [3,4]. Yet the higher education context remains less clearly synthesised, despite being a period in which communication demands, autonomy, academic pressure, identity development, and help-seeking expectations converge.

This gap matters because university students already experience substantial mental health vulnerability. In the WHO World Mental Health International College Student project, 31% of full-time first-year students across 19 colleges in eight countries screened positive for at least one 12-month mental disorder [5]. For students with developmental communication disorders, mental health risks may be intensified by communication-specific stressors, including fear of negative evaluation, avoidance of oral tasks, difficulties approaching staff, uncertainty about disclosure, and repeated experiences of being misunderstood. These pressures may affect wellbeing, academic participation, peer relationships, and sense of belonging.

Research on disability and inclusion in higher education further shows that access is not secured by enrolment alone. Students with disability, medical, and mental health conditions continue to face barriers related to aspirations, access, accommodations, and participation [6]. Accommodation systems may also place responsibility on students to disclose, self-advocate, and repeatedly prove need while leaving inflexible teaching and assessment practices largely unchanged [7]. These issues are especially relevant to communication disorders because students’ difficulties may be hidden, context-dependent, or misread as anxiety, poor preparation, low confidence, weak motivation, or limited academic ability.

Despite these intersecting concerns, evidence on developmental communication disorders in higher education remains fragmented. Existing studies have examined students who stutter [8,9,10,11,12,13,14], students with DLD or language/literacy-related profiles [15,16,17,18,19], students with cluttering characteristics [20,21], and students with broader communication-related participation needs [22,23]. Other studies have focused on instructor perceptions, disclosure, accommodations, disability information, or culturally specific expectations around speaking and participation [24,25,26,27]. However, this evidence has not yet been synthesised to explain how these profiles intersect with mental health, academic participation, and social belonging in higher education.

Because terminology in higher education research is inconsistent, this review included both core developmental communication disorders and adjacent communication-related developmental profiles. Adjacent profiles, such as developmental dyslexia, specific learning disabilities, ASD-related social communication difficulties, and nonverbal learning disability, were included only when communication participation, language/literacy functioning, academic communication demands, or social belonging were central to the study. This boundary was used to avoid treating all developmental or learning conditions as communication disorders while still capturing studies that were directly relevant to communication-based participation in higher education.

This scoping review was guided by four questions: (1) What developmental communication disorders and communication-related developmental profiles have been studied among tertiary students? (2) What mental health, academic participation, and social belonging outcomes have been reported? (3) What institutional, cultural, and support-related factors shape these students’ experiences? (4) What methodological limitations and evidence gaps remain in the literature? By synthesising this evidence, the review aims to inform more integrated approaches to student support across inclusive teaching, disability services, counselling, speech–language pathology, and institutional policy.

## 2. Methods

This study was designed to map empirical evidence on developmental communication disorders and communication-related developmental profiles among students in higher education, focusing on mental health, academic participation, social belonging, and contextual factors shaping access to support. A scoping review approach was selected because the evidence base was heterogeneous, spanning different communication profiles, study designs, outcome measures, and higher education contexts. The review was guided by the Preferred Reporting Items for Systematic Reviews and Meta-Analyses Extension for Scoping Reviews (PRISMA-ScR [28]) and informed by the Joanna Briggs Institute (Adelaide University, Australia) guidance for scoping reviews, which emphasises transparent eligibility criteria, systematic study selection, and structured data charting [29].

### 2.1. Eligibility Criteria

Eligibility criteria were developed using a PECO-informed framework, covering population, exposure, comparator, and outcomes. The population was limited to students enrolled in tertiary or higher education, including university, college, undergraduate, postgraduate, and graduate students. The exposure of interest was developmental communication disorders or communication-related developmental profiles. Core developmental communication disorders included DLD, language impairment, specific language impairment, developmental stuttering, fluency disorders, cluttering, speech sound disorder, social communication disorder, pragmatic language impairment, and clearly documented histories of these conditions.

Because higher education research does not always use clinical communication-disorder terminology consistently, the review also considered adjacent communication-related developmental profiles. Studies involving developmental dyslexia, specific learning disabilities, ASD-related social communication difficulties, or nonverbal learning disability were included only when communication participation, language/literacy functioning, academic communication demands, or social belonging were central to the study aim, measures, or findings. This operational boundary was used to manage heterogeneity and reduce conceptual drift.

Comparator groups were not required because the review included qualitative, descriptive, and single-group studies. Where studies included comparison groups, this information was extracted. Outcomes of interest included mental health, academic functioning, social participation, belonging, disclosure, accommodation, and help-seeking. The criteria were designed to map communication-related participation needs in higher education, rather than to estimate the prevalence or effect size of any single disorder-outcome relationship.

Studies were included if they met all of the following criteria: participants were tertiary, college, or university students; the study focused on a developmental speech, language, fluency, or communication disorder, or a communication-related developmental profile as defined above; at least one relevant mental health, academic, or social participation outcome was reported; the study used an empirical quantitative, qualitative, mixed-methods, intervention, case study, autoethnographic, or audit/content-analysis design; the article was peer-reviewed; and the article was published in English from 2000 onwards.

Studies were excluded if they focused only on school-aged children, non-student adult populations, acquired communication disorders such as aphasia or dysarthria, or adjacent conditions without separable communication-disorder or communication-participation data. Editorials, commentaries, conference abstracts, protocols, and studies addressing general communication difficulty without a defined developmental communication profile were also excluded. Table 1 summarises the eligibility criteria.

### 2.2. Search Strategy

A systematic search was conducted across five databases: PubMed, PsycINFO, CINAHL, Scopus, and Web of Science. These databases were selected to capture evidence across health, psychology, allied health, education, and interdisciplinary research. The search combined three concept blocks: higher education; developmental communication disorders or communication-related developmental profiles; and mental health, academic or social participation outcomes. Table 2 summarises the research structure. Full database-specific search strings are provided in Supplementary Table S1.

Searches were conducted between February and March 2026. Search results were exported to Covidence for duplicate removal and screening. Reference lists of included articles were also checked to identify additional relevant studies.

### 2.3. Study Selection

Study selection occurred in two stages. First, titles and abstracts were screened against the eligibility criteria in Covidence. The screening process was conducted by two reviewers, with an initial calibration stage used to check consistency in applying the inclusion and exclusion criteria. Disagreements were resolved through discussion, with a third reviewer consulted where consensus could not be reached. Full-text screening was then conducted using the same eligibility criteria, and reasons for exclusion were recorded in Covidence.

**Figure 1:**
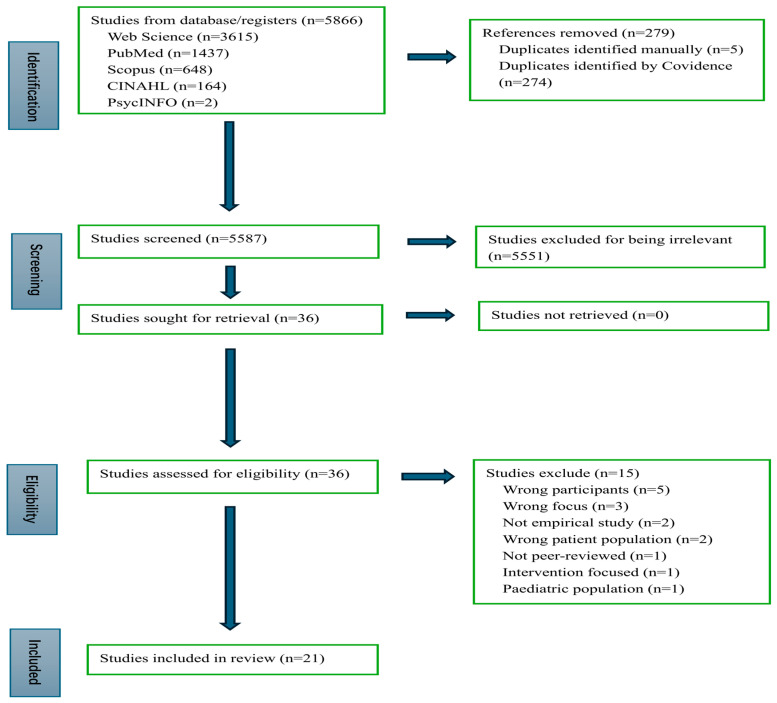
PRISMA flow diagram of study selection.

A total of 5866 records were identified through database searching. After duplicate removal, 5587 records proceeded to title and abstract screening. Of these, 36 full-text articles were assessed for eligibility, and 21 studies were included in the final review. Reasons for exclusion at the full-text stage included wrong participants, wrong focus, not empirical, not peer-reviewed, intervention/outcome focus outside the review scope, paediatric population, or wrong patient population. The study selection process is reported in Figure 1.

### 2.4. Data Charting

Data were charted using a structured extraction form developed to align with the review questions. Extracted information included author, year, country, study aim, design, participant characteristics, sample size, higher education context, communication-disorder or communication-related profile, method of identifying the disorder or communication need, comparator group where applicable, mental health outcomes, academic outcomes, social participation outcomes, key findings, limitations, and implications for higher education support. Charted data were checked for accuracy and consistency before synthesis. This approach is consistent with JBI guidance that scoping review data charting captures information relevant to the review objective, including participants, concept, context, methods, and key results [29] (see Table 3 for details).

Because the included evidence was expected to vary substantially in design and terminology, data charting was both descriptive and interpretive [30]. Descriptive extraction captured study characteristics and reported findings, while interpretive charting identified how each study contributed to the broader review domains of mental health, academic participation, social belonging, disclosure, accommodation, support access, and cultural or institutional context. All 21 included studies were mapped against these domains to ensure that the synthesis represented the full evidence base rather than selectively foregrounding only the most frequently studied conditions.

### 2.5. Quality Appraisal

Although critical appraisal is not always required in scoping reviews, a quality appraisal was conducted to support transparent interpretation of the evidence. Because the included studies used varied designs, appraisal was conducted using CASP-informed domains adapted to study type [31]. Qualitative studies were assessed for methodological fit, recruitment, reflexivity, ethics, analytic rigour, clarity of findings, and relevance to the review question. Quantitative, experimental, intervention, case study, and audit studies were assessed for clarity of aims, sampling, measurement, design appropriateness, analytic rigour, ethics, clarity of findings, and relevance.

The appraisal was used to guide interpretation rather than to exclude studies. Appraisal ratings therefore indicate the credibility and usefulness of each study in answering the review questions within its own design, not a direct ranking across study types. Table 4 summarises the appraisal approach, and Table 5 summarises the appraisal findings for individual studies.

Quality appraisal findings were reported descriptively. Particular attention was given to recurring limitations, including reliance on self-report, small or specialised samples, inconsistent diagnostic confirmation, limited longitudinal evidence, and uneven representation of different developmental communication disorders and communication-related profiles.

### 2.6. Data Synthesis

A narrative and thematic synthesis was conducted as the included studies varied in design, population, communication-disorder focus, and outcome measurement. The synthesis proceeded in three stages. First, studies were summarised descriptively according to year, country, study design, participant group, and communication-disorder profile. Second, findings were mapped to the review’s core outcome domains: mental health and emotional wellbeing, academic functioning and participation, social belonging and peer relationships, disclosure and stigma, accommodations and support access, and cultural or institutional context. Finally, cross-study patterns were synthesised to identify how developmental communication disorders function as hidden communication needs in higher education.

The synthesis was organised to distinguish reported findings from broader interpretation. Section 3, therefore, presents evidence patterns across the 21 studies, while the Discussion interprets these patterns in relation to current debates on hidden disability, communication access, inclusive higher education, student mental health, and interdisciplinary support. This is purposefully followed to avoid overextending claims beyond the evidence while still allowing the review to generate a coherent conceptual contribution.

## 3. Results

### 3.1. Study Characteristics

The 21 included studies were published between 2000 and 2025, showing a small but growing evidence base. The studies were conducted across a range of countries, although the distribution was uneven. Nine studies were from the United States [8,11,12,13,14,15,16,23,24]. Three studies were from Israel [17,20,21]. The remaining studies were from Bangladesh [32], South Africa [10], Belgium/Flanders [22], Australia [25], Poland [26], Canada [18], and China [19,27]. One study compared the experiences of students from China and Japan [9].

The studies used varied methods. Qualitative designs included interpretative phenomenological analysis, semi-structured interviews, narrative inquiry, case study, and autoethnography [8,9,10,11,27,32]. Two studies used mixed methods, combining survey data with qualitative responses [12,24]. Several studies used quantitative observational, comparative, or cross-sectional designs [15,16,17,19,20,21,22]. Others used experimental, quasi-experimental, or intervention designs [13,14,18,26]. Meredith [25] used a website audit/content analysis to examine disability information for students who stutter.

The evidence was broad but uneven. Stuttering and fluency-related experiences were the most commonly studied, including students who stutter, women who stutter, instructor perceptions, disclosure, oral presentations, co-curricular participation, and cross-cultural experiences of stuttering [8,9,10,11,12,13,14,24,25,26,27]. A smaller group of studies examined DLD, language impairment, dyslexia, specific learning disorder, reading and spelling difficulties, and phonological or morphological processing in university students [15,16,17,18,19]. Two studies examined self-identified cluttering characteristics [20,21]. One study focused on ASD-related social communication and participation difficulties [22], and one case study examined Russell–Silver syndrome with a nonverbal learning disability profile involving academic, communication, and social–emotional implications [23]. Table 3 (see Section 2.4) summarises main evidence clusters represented in the review. Full study characteristics are provided in Supplementary Table S2.

### 3.2. Methodological Quality

The studies reviewed provided useful and relevant evidence for mapping developmental communication disorders and communication-related participation needs in higher education. Nineteen studies were appraised as high-value contributions to the review question, while two were appraised as medium-value contributions. Most studies had clear aims, appropriate overall designs, relevant data collection methods, and clearly reported findings. However, the evidence base also had several recurring limitations. These included reliance on self-report for identifying communication conditions, small or specialised samples, limited diagnostic confirmation, restricted generalisability, and few longitudinal or controlled intervention studies.

Several qualitative studies provided rich accounts of lived experience, particularly in relation to stuttering, identity, disclosure, stigma, and university participation [8,9,10,11,27]. However, reflexivity was inconsistently reported across qualitative and mixed-methods studies. Quantitative studies generally used appropriate survey, experimental, or standardised assessment designs, but several relied on cross-sectional data, limiting causal interpretation [15,19,20,21,22]. Intervention evidence was especially limited. Spigarelli [18], for example, provided promising pre–post evidence for a morphological and phonological awareness intervention among university students with specific reading and learning disabilities, but the study did not include a control group or follow-up assessment. Experimental vignette studies by Werle and Byrd [13,14] and Otrębski [26] also offered controlled evidence about perceptions of stuttering, self-disclosure, communication competence, and attitudes, but these studies necessarily relied on simulated rather than naturally occurring classroom interactions. Table 5 (See Section 2.5) summarises the appraisal findings by evidence type. Full study-level appraisal details are provided in Supplementary Table S3.

### 3.3. Thematic Synthesis

Five interrelated themes were identified across the 21 included studies: mental health and emotional wellbeing; academic functioning and participation; social belonging, stigma, and disclosure; institutional support and accommodations; and cultural and contextual influences. These themes overlapped substantially. Mental health concerns were often embedded in academic communication demands, while academic and social participation were shaped by stigma, disclosure, institutional responses, and cultural expectations. Table 6 summarises the main thematic patterns and evidence gaps.

#### 3.3.1. Mental Health and Emotional Well-Being

Across the studies, mental health difficulties were rarely presented as isolated individual symptoms. Instead, anxiety, stress, depression, psychosomatic symptoms, reduced self-efficacy, and lower wellbeing were consistently linked to communicative visibility, academic evaluation, stigma, and uncertainty about support. This pattern was clearest in studies of oral presentation and public speaking. Ahmed [32] and Zong [27] both showed that presentation-related anxiety was intensified by fear of judgment, English-medium demands, limited confidence, and classroom evaluation. Although these studies were conducted in different national contexts, they converge in showing that stuttering or stuttering-like disruptions were embedded in broader performance conditions rather than operating as purely speech-based difficulties.

Stuttering-focused studies similarly linked psychological distress to the social meaning of fluency. It was showed that distress was shaped by anticipation, negative evaluation, gendered assumptions, cultural norms, disclosure decisions, and repeated pressure to communicate quickly and confidently [8,9,10,11,12]. However, these studies were largely qualitative or retrospective, meaning they provide rich evidence of lived experience but limited capacity to establish directionality between stuttering, participation barriers, and mental health outcomes.

Quantitative studies on cluttering and learning-related profiles extended the mental health evidence beyond stuttering. Icht et al. [20] and Zukerman et al. [21] found that students who self-identified with cluttering characteristics reported poorer well-being indicators, including depression, psychosomatic symptoms, stress, and self-inefficacy. Heiman and Precel [17] reported higher exam-related stress among students with learning disabilities, while Wang et al. [19] showed that state anxiety interacted with working memory in predicting academic performance among students with specific learning disabilities. Plotts and Livermore [23] also identified mild depression within a case involving Russell–Silver syndrome and a nonverbal learning disability profile. These findings suggest a broader relationship between communication-related developmental profiles and mental health vulnerability, although several studies relied on self-report, cross-sectional designs, or heterogeneous disability categories.

#### 3.3.2. Academic Functioning and Participation

The reviewed studies suggest that academic participation is a more useful concept than academic performance alone. Some studies examined conventional academic outcomes, including reading, spelling, working memory, foreign-language learning, and GPA-related indicators [15,16,17,18,19]. These studies showed that developmental language, literacy, and learning-related profiles can continue to affect university-level academic functioning. Del Tufo and Earle [15], for example, distinguished the cognitive–language profiles of students with histories of DLD and developmental dyslexia. These findings suggest that different developmental language and literacy profiles may require different academic supports, rather than a generic accommodation model [33]. Downey et al. [16] also showed persistent phonological processing difficulties affecting foreign-language learning. Spigarelli et al. [18] further provided preliminary intervention evidence that targeted morphological and phonological awareness support may improve selected literacy outcomes in university students with developmental dyslexia and dysgraphia.

However, the evidence also shows that academic difficulty is not reducible to grades or test scores. For students who stutter, oral assessment emerged as a recurring site of vulnerability. Daniels et al. [24], Werle and Byrd [12,13,14], Sasso et al. [11], Isaacs [10], Ahmed [32], and Zong [27] collectively show that oral presentations are shaped by instructor perceptions, fluency expectations, communication competence, self-disclosure, fear of evaluation, and the pace of academic interaction. A key tension appears here: experimental evidence suggests that self-disclosure and communication competence can improve instructor ratings [13,14], while qualitative evidence shows that disclosure and oral performance can also create emotional labour, stigma, and additional risk [8,10,11].

The findings, therefore, point to academic participation as an interaction between student profiles and institutional design. Modified foreign-language instruction supported dyslexic students in Downey et al. [16], while individually matched accommodations were important in Jansen et al. [22]. At the same time, support was often limited when academic tasks required rapid verbal response, public performance, or self-advocacy. The evidence base remains uneven: language and literacy-related studies provide stronger cognitive–academic measurement, whereas stuttering studies provide richer evidence of oral participation and assessment inequity. Few studies combine both academic outcome measurement and lived-experience data, leaving a gap in understanding how academic performance, participation, and mental health interact over time.

#### 3.3.3. Social Belonging, Stigma, and Disclosure

Social belonging emerged as a central mechanism linking communication disorders with both mental health and academic participation. The clearest evidence came from stuttering-focused studies. It was shown that students who stutter navigated peer judgement, instructor expectations, microaggressions, gendered assumptions, cultural norms, and concerns about how their communication would be interpreted [8,9,10,11,27]. These experiences affected not only classroom participation but also identity, relationships, and co-curricular involvement.

Disclosure was one of the most complex and inconsistent areas in the evidence. Werle and Byrd [14] found that self-disclosure of stuttering improved positive instructor ratings, suggesting that disclosure may reduce some negative perceptions in controlled assessment contexts. Yet qualitative studies showed that disclosure is not necessarily protective. Students may disclose into environments where staff lack knowledge, peers hold stereotypes, or support pathways are unclear [8,9,10,11]. Meredith et al. [25] further showed that university disability information for students who stutter was often difficult to locate, meaning that students may be expected to disclose before knowing whether the institution is prepared to respond appropriately.

Attitudinal evidence also showed that stigma operates through both peers and instructors. Daniels et al. [24] found that instructors varied in their understanding of stuttering and classroom support. Otrębski et al. [26] found that negative feelings toward a peer who stutters were associated with less positive attitudes, with gender and academic major shaping responses. Werle and Byrd [12,13,14] further showed that perceived competence, fluency, and disclosure could influence instructor evaluations. These findings suggest that participation barriers are not only caused by the communication disorder itself but also by how others interpret communication differences.

Evidence beyond stuttering was more limited but still relevant. Jansen et al. [22] found that students with ASD-related social communication difficulties experienced barriers that could not be fully addressed through formal accommodations alone. Plotts and Livermore [23] similarly highlighted the social–emotional implications of a nonverbal learning disability profile. Together, these studies suggest that social belonging requires more than individual adjustment; it depends on whether communication differences are recognised, understood, and accommodated within everyday academic and social environments.

#### 3.3.4. Institutional Support and Accommodations

The evidence consistently indicates that higher education support systems are important but incomplete. Some studies showed that accommodations and targeted supports can improve participation. Downey et al. [16] reported that modified foreign-language instruction supported students with dyslexia, while Spigarelli et al. [18] suggested that speech–language and literacy-informed intervention may remain relevant in higher education. Heiman and Precel [17] highlighted the importance of study strategies and special test conditions, and Jansen et al. [22] showed that accommodations such as extended exam duration, exam deferral, and smaller exam groups could be useful for some students.

At the same time, formal accommodations did not address all communication-related barriers. Jansen et al. [22] found that social communication problems often required coaching or therapy beyond exam adjustments. Sasso et al. [11] showed that students who stutter continued to experience exclusion in co-curricular and interpersonal spaces, where formal accommodations were less available. Isaacs [10] similarly showed that university time structures and expectations around fast, fluent communication could remain exclusionary even when students were academically capable. These findings suggest that accommodation systems are necessary but insufficient when institutional norms themselves privilege particular forms of communication.

Institutional visibility also emerged as a barrier. Meredith et al. [25] found that Australian university websites provided limited information about support for students who stutter, including alternative teaching and assessment arrangements. This matters because support-seeking often depends on whether students can identify themselves as eligible for assistance and whether institutions signal that communication disorders are recognised. Across studies, staff knowledge was another recurring concern. Daniels et al. [24] and Werle and Byrd [12,13,14] showed that instructor perceptions and assessment practices can shape students’ participation, while Azios et al. [8] and He et al. [9] showed that clinicians, counselling, disability services, and authority figures may either support or constrain students’ university experiences.

The key synthesis is that support needs to move from reactive accommodation to communication-accessible systems. This includes visible disability information, staff training, flexible assessment, explicit oral-task scaffolding, counselling pathways, speech–language pathology input, and academic language/literacy support. However, very few included studies evaluated such integrated models, leaving a major gap between identified need and tested support.

#### 3.3.5. Cultural and Contextual Influences

Several studies showed that communication disorders in higher education are shaped by linguistic, cultural, and institutional context. Ahmed [32] and Zong [27] demonstrated that English-medium presentations and second-language academic performance can intensify communication anxiety. Downey et al. [16] and Heiman and Precel [17] similarly showed that foreign-language learning can be especially demanding for students with dyslexia or learning disabilities. These studies suggest that communication-related vulnerability may be amplified when students must demonstrate academic competence through an additional language or in contexts where oral performance is strongly evaluated.

Cross-cultural evidence further complicates assumptions about disclosure and support. He et al. [9] found that Chinese and Japanese students who stutter shared experiences of communication barriers and psychological impact, but differed in coping preferences and perceived support needs. Zong [27] linked presentation anxiety among Chinese postgraduates to cultural expectations, fear of negative evaluation, and classroom interaction. Otrębski et al. [26] showed that attitudes toward a peer who stutters were shaped by emotional responses, gender, and academic major in Poland. Isaacs [10], situated in South African higher education, showed how institutional time norms could disadvantage students who communicate differently.

These studies suggest that communication disorders are not culturally neutral experiences. Norms around face, fluency, public performance, self-advocacy, disability disclosure, and help-seeking shape whether students interpret their difficulties as support needs, whether they seek assistance, and how others respond. However, the evidence base remains limited, with few studies directly comparing cultural contexts or examining CALD, multilingual, or international student experiences. This is an important gap, given the increasingly international and multilingual nature of higher education.

## 4. Discussion

We mapped 21 empirical studies on developmental communication disorders and communication-related developmental profiles in higher education. The evidence was diverse in design, country, and disorder focus. It was strongest for stuttering and fluency-related participation, with smaller bodies of evidence on DLD/language impairment, dyslexia and language-related learning profiles, cluttering, ASD-related social communication, and nonverbal learning disability. Across this evidence base, the reviewed studies suggest that communication disorders and related profiles can become participation vulnerabilities when university systems require students to show competence through rapid, fluent, public, and socially confident communication.

This finding is important because higher education is already a setting of substantial mental health need. In the WHO World Mental Health International College Student project, 31% of full-time first-year students screened positive for at least one 12-month mental disorder [5]. Other WHO work has shown that mental disorders among college students are often untreated and that pre-matriculation mental disorders are associated with later college attrition [34]. The present review suggests that developmental communication disorders may add a specific layer of risk. This risk does not arise from communication difficulty alone. It is shaped by repeated encounters with academic, social, and institutional environments that are not designed for communication diversity.

The findings support viewing higher education as a communication-intensive ecology. Students are not only required to understand the course content. They also need to ask questions, contribute to discussions, complete oral presentations, approach lecturers, work with peers, disclose support needs, and take part in formal and informal learning spaces. For students with developmental communication disorders, these everyday tasks can become sources of stress, judgment, and extra effort.

This was most visible in stuttering-focused studies. Oral presentations, instructor ratings, self-disclosure, peer attitudes, time pressure, and co-curricular participation repeatedly shaped students’ experiences [8,9,10,11,12,13,14,24,26,27]. Similar patterns were also found in studies of DLD, dyslexia, learning-related language profiles, cluttering, ASD-related social communication, and nonverbal learning disability. In these studies, difficulties were linked to reading, working memory, foreign-language learning, assessment, self-efficacy, help-seeking, and access to accommodations [15,16,17,18,19,20,21,22,23].

A key contribution of this review is therefore to move beyond a narrow impairment model. A student who stutters may be academically capable but disadvantaged when oral fluency and speed are treated as signs of competence. A student with DLD or dyslexia may understand course content but struggle when teaching and assessment rely heavily on rapid language processing, dense written information, or foreign-language requirements. A student with cluttering characteristics may experience reduced well-being without ever being identified for speech–language support. A student with social communication difficulties may receive exam adjustments but remain unsupported in tutorials, group work, and help-seeking. In each case, the difficulty lies in the interaction between the student’s communication profile and the way higher education is designed around communication.

Mental health findings should also be read through this interactional lens. Across the included studies, anxiety, stress, depression, psychosomatic symptoms, self-efficacy, and reduced well-being were often linked to the conditions in which students had to communicate. These included fear of negative evaluation, public speaking, stigma, limited preparation, language barriers, unclear accommodations, and uncertainty about disclosure [8,9,10,11,12,17,19,20,21,27,32].

This interpretation is consistent with research on adults with DLD, which shows that poor mental health may be shaped by masking, exhaustion, social disconnection, discrimination, and lack of appropriate support [2]. The reviewed evidence does not show that communication disorders directly cause mental health difficulties. Rather, it shows that mental health risks often emerge through the interaction between students’ communication profiles and the environments in which they study, speak, disclose, and seek help.

This distinction is important. Several studies showed links between communication demands and emotional burden, but many relied on qualitative, cross-sectional, retrospective, or self-report designs. The two cluttering studies identified associations between self-identified cluttering characteristics and poorer wellbeing indicators, but they did not use clinical diagnosis or longitudinal methods [20,21]. Similarly, stuttering studies gave rich evidence of lived experience, but they could not always establish whether mental health difficulties preceded, followed, or developed alongside communication-related exclusion. These limits do not weaken the review’s main message. They clarify what the field can and cannot yet claim.

Academic participation also emerged as broader than academic performance. Some studies examined traditional outcomes such as GPA, reading, spelling, working memory, foreign-language learning, and intervention-related literacy gains [15,16,17,18,19]. Yet many of the most important barriers were not captured by grades alone. They involved oral presentations, feedback-seeking, group work, disclosure, co-curricular participation, and how others judged students’ competence. Students may therefore appear successful while still paying a high emotional and communicative cost. Sasso et al. [11] described this as an “academic oratory tax”, while Isaacs [10] showed how university time norms can privilege speed and fluency. These findings align with broader work showing that assessment accommodations may support access while still leaving assessment systems that privilege non-disabled norms largely unchanged [7].

Disclosure was one of the clearest areas of tension in the evidence. Werle and Byrd [14] found that self-disclosure of stuttering could improve instructor ratings, especially when communication competence was lower. However, qualitative studies showed that disclosure can also expose students to stigma, misunderstanding, lower expectations, or the burden of educating others [8,9,10,11]. Meredith et al. [25] further showed that university disability information for students who stutter was often limited. This means students may be expected to disclose to systems that do not clearly show whether their needs will be understood. The same problem has been noted in wider disability research, where students often seek full membership in university life but face stigma, inconsistent support, and repeated self-advocacy demands [6].

The evidence also shows that accommodation-based inclusion is necessary but not sufficient. Extra time, modified instruction, exam adjustments, and targeted literacy support can help some students [16,17,18,22]. However, formal adjustments do not fully address stigma, oral-performance norms, weak disability information, instructor uncertainty, or exclusion from co-curricular spaces [11,12,13,14,24,25]. Universities therefore need to move from reactive accommodation toward communication-accessible systems. This may include clear disability information, staff training, flexible assessment, explicit support for oral tasks, accessible help-seeking pathways, counselling links, speech–language pathology input, and academic language and literacy support. Universal Design for Learning also offers a useful framework for reducing mismatches between students and instructional design, although individual supports will still be needed for some students [35,36].

Cultural and linguistic context further shaped students’ experiences. Ahmed [32], He et al. [9], and Zong [27] showed that public speaking, English-medium instruction, fear of negative evaluation, cultural expectations, and help-seeking norms influenced how students experienced speech disruption, stuttering, and presentation anxiety. Downey et al. [16] and Heiman and Precel [17] also showed that foreign-language learning can be difficult for students with dyslexia or learning disabilities. These findings are important because higher education is increasingly multilingual and international. They also caution against assuming that disclosure, counselling, self-advocacy, or classroom participation operate in the same way across contexts. More culturally responsive research is needed, particularly with CALD students, international students, and students in non-Western higher education systems.

This review has practical implications for higher education. Universities should recognise developmental communication disorders and communication-related profiles as potential hidden support needs that can affect mental health, academic participation, and belonging. This recognition should be visible in disability service information, counselling intake processes, staff development, and academic support pathways. Oral communication tasks should also be reviewed through an inclusive assessment lens. When oral communication is a core learning outcome, students should receive clear criteria, preparation time, explicit teaching, and appropriate support. When oral delivery is not central to the outcome, alternative formats should be considered. Support should not depend only on students’ willingness or ability to disclose. Clear information, inclusive teaching, and staff awareness can reduce the burden of repeated self-advocacy.

The evidence also points to the need for stronger interdisciplinary pathways. Students who stutter may benefit from communication-specific strategies as well as psychologically safe disclosure planning. Students with DLD or dyslexia may need language/literacy-informed academic support. Students with cluttering characteristics may need pathways that connect communication concerns with wellbeing support. Students with social communication difficulties may need coaching that goes beyond exam accommodations. These needs sit across speech–language pathology, counselling, disability advising, teaching teams, academic language and learning support, and peer support. Without coordination, students may be passed between services that each address only part of the problem.

Several limitations should be acknowledged. This review was limited to English-language, peer-reviewed journal articles published from 2000 onwards, so relevant studies in other languages or grey literature may have been missed. The included studies also varied in design, terminology, population, and outcome measurement. This level of variation is expected in a scoping review, but it limits what can be said about the strength or size of links between communication disorders, mental health, academic participation, and social belonging.

The evidence base itself was also uneven. Stuttering and fluency-related studies were overrepresented, while direct higher education research on DLD, speech sound disorder, pragmatic language impairment, and mixed developmental communication profiles was limited. Many studies relied on self-report, retrospective accounts, cross-sectional designs, vignette-based experiments, small samples, or self-identified communication characteristics. These designs provided useful evidence about experience, perception, and participation, but they offered limited support for causal claims. The findings should therefore be read as a map of current evidence and gaps, not as proof of direct cause-and-effect relationships.

Future research should address these gaps directly. Longitudinal studies are needed to examine how communication needs affect transition into university, academic progression, retention, placement participation, mental health, social belonging, and transition into employment. More research is needed on DLD, speech sound disorder, pragmatic language impairment, cluttering, and mixed communication profiles among university students. Studies should use clearer diagnostic criteria where possible, while recognising that many adults enter higher education with undiagnosed or historically under-identified communication needs. Intervention research is also needed to test communication-accessible teaching, flexible assessment, staff training, peer support, counselling–disability service pathways, and speech–language pathology involvement in higher education. This would move the field beyond documenting barriers toward evaluating support models that make universities more accessible for students with hidden communication needs.

## 5. Conclusions

This scoping review mapped 21 empirical studies on developmental communication disorders and communication-related developmental profiles in higher education. Across the evidence base, students’ difficulties were not limited to speech, language, fluency, literacy, or social communication in isolation. They became most consequential when they interacted with communication-intensive university demands, including oral presentations, tutorials, group work, foreign-language learning, assessment, help-seeking, disclosure, and co-curricular participation.

The review suggests that developmental communication disorders and related communication profiles can function as hidden communication needs that create participation vulnerabilities across mental health, academic functioning, and social belonging. Anxiety, stress, depression, self-inefficacy, stigma, reduced confidence, and exclusion were often linked to institutional and social conditions, including fluency norms, fear of negative evaluation, limited staff awareness, unclear accommodation pathways, and culturally shaped expectations around communication.

These conclusions should be read in light of an uneven evidence base. The strongest evidence came from stuttering and fluency-related studies, while direct evidence on DLD, speech sound disorder, pragmatic language impairment, and mixed developmental communication profiles remains limited. Even so, the review shows that higher education needs communication-accessible systems that anticipate diverse speech, language, fluency, literacy, and pragmatic communication profiles. This includes flexible assessment, explicit support for oral communication tasks, visible disability information, staff training, culturally responsive practice, and stronger pathways between teaching teams, disability services, counselling, academic language and literacy support, and speech–language pathology.

By bringing together evidence across mental health, academic participation, social belonging, and institutional support, this review highlights higher education as an important but under-recognised site for lifespan communication-disorder research and practice. Supporting students with hidden communication needs is not only a matter of individual adjustment. It is also a matter of designing universities where communication diversity is anticipated, understood, and valued.

## Figures and Tables

**Table 1:** PECO framework and eligibility criteria.

PECO Domain	Inclusion Criteria	Exclusion Criteria	Operational Decision Rule
*Population*	Students enrolled in tertiary or higher education, including university, college, undergraduate, postgraduate, or graduate students.	School-aged students; non-student adult populations; clinical samples not linked to higher education.	Include only when participants are explicitly situated in a higher education context. Studies with mixed samples are included only if tertiary student data are separately reported or clearly interpretable.
*Exposure*/ *condition*	Developmental communication disorders or communication-related developmental profiles, including DLD, language impairment, specific language impairment, developmental stuttering, fluency disorder, cluttering, speech sound disorder, social communication disorder, pragmatic language impairment, or documented history of these conditions. Adjacent profiles were included only where communication participation, language/literacy functioning, academic communication demands, or social belonging were central.	Acquired communication disorders, including aphasia, dysarthria, acquired apraxia of speech, or communication difficulties secondary to adult-onset neurological injury. Adjacent developmental or learning conditions were excluded where communication-disorder or communication-participation data were not separable.	Include studies where speech, language, fluency, pragmatic communication, language/literacy functioning, or communication-related participation is central to the study aim, population, measures, or findings.
*Comparator*	Any comparator group, including students without communication disorders, typically developing peers, students with other disabilities, or no comparator.	Not applicable.	Comparator groups are not required because qualitative, descriptive, and single-group studies are eligible. Where comparators are used, their characteristics are extracted.
*Primary outcomes*	Mental health and emotional wellbeing outcomes, including anxiety, depression, social anxiety, psychological distress, stress, wellbeing, loneliness, self-esteem, self-efficacy, psychosomatic symptoms, or emotional burden.	Studies with no mental health, wellbeing, academic, or social participation outcome.	Include where mental health or wellbeing is measured directly or discussed as a substantive finding.
*Secondary outcomes*	Academic functioning and academic participation, including academic performance, GPA, grades, retention, attrition, assessment participation, oral presentations, classroom participation, foreign-language learning, help-seeking, accommodation use, or academic self-efficacy.	Studies focused only on linguistic performance without any higher education participation, academic, mental health, or social relevance.	Include studies that examine academic participation broadly, not only formal achievement indicators.
*Social participation outcomes*	Social belonging, peer relationships, disclosure, stigma, identity, social integration, co-curricular participation, communication confidence, or perceived instructor/peer attitudes.	Studies addressing general communication preferences without a defined developmental communication profile or a relevant participation outcome.	Include where social participation is reported as a finding, experience, barrier, facilitator, or measured outcome.
*Study design*	Empirical quantitative, qualitative, mixed-methods, experimental, quasi-experimental, intervention, case study, autoethnographic, or audit/content-analysis studies.	Editorials, commentaries, opinion papers, conference abstracts, protocols, and non-empirical papers.	Include empirical studies that contribute data relevant to the review questions.
*Publication type*	Peer-reviewed journal articles.	Grey literature, dissertations, theses, book chapters, non-peer-reviewed reports, and conference proceedings.	Restricting to peer-reviewed articles supports quality and feasibility while maintaining a coherent evidence base.
*Language*	English-language publications.	Non-English publications.	Non-English studies are excluded unless a later bilingual extension is conducted.
*Publication period*	Studies published from 2000 onwards.	Studies published before 2000.	The review focuses on contemporary higher education, disability support, and student mental health contexts.

**Note.** The eligibility criteria were developed to capture empirical studies of tertiary students with developmental communication disorders or communication-related developmental profiles, focusing on mental health, academic participation, and social belonging. The criteria reflect the final review scope and search strategy.

**Table 2:** Database search strategy.

Concept Block	Search Purpose	Example Terms	Full Search String
*High education*	Identify tertiary or university contexts	university student *, college student *, undergraduate *, postgradu-ate *, graduate student *, higher education, tertiary education	Supplementary Table S1
*Communication disorders*/*profiles*	Identify core developmental communication disorders and adjacent communication-related profiles	developmental language disorder, DLD, language impairment, stutter *, stammer *, fluency disorder *, cluttering, speech disorder *, pragmatic language, dyslexia, specific learning disability *	Supplementary Table S1
*Outcomes*	Identify mental health, academic, and social participation outcomes	anxiety, depression, wellbeing, academic performance, participation, belonging, disclosure, accommodation, help-seeking	Supplementary Table S1

**Note.** 1. The search strategy combined three concept blocks: higher education, developmental communication disorders or communication-related developmental profiles, and mental health, academic, or social participation outcomes. The core Boolean logic was higher education AND developmental communication disorders AND mental health/academic/social participation outcomes. This aligns with the final PECO-informed scope of the review. 2. In this table, an asterisk (*) is placed beside a term to indicate that it serves as a core or pivot keyword. It indicates a term that appears repeatedly across multiple search contexts or that exhibits semantic overlap with other concept blocks.

**Table 3:** Evidence clusters represented in the included studies.

Evidence Cluster	Studies	Main Focus	Evidence Balance
Stuttering and fluency-related participation	Azios et al. (2022) [8]; He et al. (2025) [9]; Isaacs (2020) [10]; Sasso et al. (2024) [11]; Werle & Byrd (2021, 2022a, 2022b) [12,13,14]; Daniels et al. (2011) [24]; Meredith et al. (2012) [25]; Otrębski et al. (2024) [26]; Zong (2025) [27]	Oral presentations, instructor and peer perceptions, disclosure, co-curricular participation, culture, and support visibility.	Largest and most coherent evidence cluster; strongest basis for claims about oral participation, disclosure, stigma, and belonging.
DLD, language/literacy, dyslexia, and specific learning profiles	Del Tufo & Earle (2020) [15]; Downey et al. (2000) [16]; Heiman & Precel (2003) [17]; Spigarelli et al. (2025) [18]; Wang et al. (2024) [19]	Language/literacy processing, working memory, foreign-language learning, academic stress, and preliminary intervention evidence.	Smaller and more heterogeneous cluster; stronger for cognitive–academic functioning than for lived experience or belonging.
Cluttering characteristics	Icht et al. (2023) [20]; Zukerman et al. (2024) [21]	Self-identified cluttering characteristics, depression, psychosomatic symptoms, stress, self-efficacy, and wellbeing.	Emerging evidence; limited by self-identification and cross-sectional design.
Social communication and nonverbal learning profiles	Jansen et al. (2016) [22]; Plotts & Livermore (2007) [23]	ASD-related social communication participation and one case of Russell–Silver syndrome with nonverbal learning disability.	Adjacent evidence; useful for communication participation but not directly generalisable to all developmental communication disorders.

**Table 4:** Design-sensitive quality appraisal approach.

Study Design	Main Appraisal Focus	How Findings Were Used	Main Caution
Qualitative and autoethnographic studies	Methodological fit, recruitment, data collection, reflexivity, ethics, analytic rigour, clarity of findings, and relevance.	Used to interpret lived experience, identity, disclosure, stigma, and participation barriers.	Rich evidence of experience, but limited ability to establish prevalence or causal direction.
Quantitative, experimental, and intervention studies	Clarity of aims, sampling, measurement, design appropriateness, analysis, ethics, clarity of findings, and relevance.	Used to interpret associations, group differences, attitudes, perceptions, and preliminary intervention effects.	Several studies were cross-sectional, vignette-based, self-report, or single-group designs.
Case study and audit/content analysis	Fit between aim and method, data source clarity, analytic transparency, and relevance to higher education support.	Used to interpret institutional visibility, accommodation access, and individual communication-related participation needs.	Findings are context-specific and not intended to be generalised statistically.

**Note.** The table is mapped to the review scope, which required included studies to involve tertiary students or higher education contexts, developmental communication disorders or clearly defined communication-related developmental profiles, and at least one mental health, academic, or social participation outcome.

**Table 5:** Summary of methodological appraisal by evidence type.

Evidence Type	Contribution to the Review	Main Strengths	Main Cautions
Qualitative, narrative, case, and autoethnographic studies	Explained lived experience, identity, disclosure, stigma, exclusion, and support needs.	Rich contextual data and strong relevance to participation and belonging.	Often small, context-specific, and limited for causal or prevalence claims. Reflexivity was not always fully reported.
Quantitative cross-sectional and comparative studies	Mapped associations among communication-related profiles, wellbeing, anxiety, academic outcomes, and cognitive–language functioning.	Larger samples in some studies and the use of standardised or structured measures.	Mostly cross-sectional or self-report, with variable diagnostic confirmation and limited causal inference.
Experimental and vignette studies	Examined perceptions, attitudes, disclosure, and communication competence in controlled conditions.	Useful for isolating the effects of disclosure and instructor or peer perception.	Simulated tasks may not predict actual classroom behaviour or long-term outcomes.
Intervention and institutional audit studies	Provided preliminary evidence on targeted literacy intervention and institutional visibility of support.	Direct relevance to support design and service access.	A limited number of studies; intervention evidence was mostly preliminary, and institutional audits did not measure student outcomes.

**Table 6:** Summary of thematic synthesis and evidence gaps.

Theme	Main Evidence Pattern	Key Inconsistency or Limitation	Implication for Synthesis
Mental health and emotional wellbeing	Anxiety, stress, depression, psychosomatic symptoms, self-efficacy, and reduced wellbeing were linked to communication visibility, public speaking, evaluation, stigma, and unclear support.	Evidence was strongest for stuttering and self-identified cluttering; fewer studies directly examined DLD, speech sound disorder, or pragmatic language impairment.	Mental health concerns should be interpreted as shaped by communication demands and environments, not as direct effects of diagnosis alone.
Academic functioning and participation	Studies reported effects on oral presentation, foreign-language learning, language/literacy processing, working memory, feedback-seeking, and assessment participation.	Academic performance measures were inconsistent, and many studies did not track long-term progression or retention.	Academic participation is broader than grades and includes the communication practices through which students demonstrate competence.
Social belonging, stigma, and disclosure	Disclosure, peer and instructor perceptions, gendered assumptions, stigma, and co-curricular participation shaped student experience.	Experimental studies suggested disclosure may improve ratings, while qualitative studies showed disclosure can also create risk and emotional labour.	Disclosure should be supported but not made the only pathway to inclusion.
Institutional support and accommodations	Support included modified instruction, exam accommodations, oral-task support, staff awareness, disability information, counselling, and language/literacy support.	Formal accommodations did not fully address stigma, oral-performance norms, social participation, or co-curricular exclusion.	Universities need communication-accessible systems, not only reactive individual adjustments.
Cultural and contextual influences	English-medium instruction, foreign-language learning, public speaking expectations, face, help-seeking norms, and institutional time pressures shaped experiences.	Few studies directly compared cultural contexts or examined CALD, multilingual, or international students.	Support models should be culturally responsive and tested in diverse higher education settings.

## Data Availability

No new data were created or analysed in this study. Data sharing is not applicable to this article.

## Supplementary Materials

The following supporting information can be downloaded at https://www.mdpi.com/article/10.3390/healthcare14121790/s1. Table S1: Database-specific search strategy; Table S2: Full study characteristics of the included studies; Table S3: Full study-level appraisal details of the included studies.
