# Supplementary material for: Hidden Communication Needs in Higher Education: A Scoping Review of Developmental Communication Disorders, Mental Health, and Academic Participation
Source: Healthcare (Basel). 2026 Jun 21;14(12):1790. doi: 10.3390/healthcare14121790 (PMC13299988; doi:10.3390/healthcare14121790)
Supplement: Supplementary file 1 [file healthcare-14-01790-s001.zip › Supplementary Table S1 Database-specific search strategy.pdf]

Supplementary Table S1. Database-specific search strategy

Database	Search field and syntax	Search string	Limits
PubMed	Title/abstract and MeSH-informed free-text search	((“Students”[Mesh] OR “Universities”[Mesh] OR “college student*”[tiab] OR “university student*”[tiab] OR undergraduate*[tiab] OR postgraduate*[tiab] OR “graduate student*”[tiab] OR “higher education”[tiab] OR “tertiary education”[tiab] OR universit*[tiab] OR college*[tiab]) AND (“Language Disorders”[Mesh] OR “Communication Disorders”[Mesh] OR “developmental language disorder”[tiab] OR DLD[tiab] OR “language disorder*”[tiab] OR “language impairment*”[tiab] OR “specific language impairment”[tiab] OR SLI[tiab] OR “communication disorder*”[tiab] OR “speech disorder*”[tiab] OR “speech sound disorder*”[tiab] OR stutter*[tiab] OR stammer*[tiab] OR “fluency disorder*”[tiab] OR “social communication disorder”[tiab] OR “pragmatic language impairment”[tiab] OR “social pragmatic communication”[tiab] OR “oral language difficult*”[tiab] OR “history of language disorder”[tiab]) AND (anxiety[tiab] OR depression[tiab] OR “social anxiety”[tiab] OR “psychological distress”[tiab] OR well-being[tiab] OR “well-being”[tiab] OR loneliness[tiab] OR “self-esteem”[tiab] OR “social withdrawal”[tiab] OR “mental health”[tiab] OR “mental disorder*”[tiab] OR “academic performance”[tiab] OR GPA[tiab] OR grade*[tiab] OR retention[tiab] OR attrition[tiab] OR dropout[tiab] OR persistence[tiab] OR “academic engagement”[tiab] OR participation[tiab] OR “social integration”[tiab] OR belonging[tiab] OR “peer relation*”[tiab] OR friendship*[tiab] OR disclosure[tiab] OR accommodation*[tiab] OR “help-seeking”[tiab]))	English; peer-reviewed journal articles; 2000 onward
PsycINFO	Title/abstract/keyword search	((“college student*” OR “university student*” OR undergraduate* OR postgraduate* OR “graduate student*” OR “higher education” OR “tertiary education” OR universit* OR college*) AND (“developmental language disorder” OR DLD OR “language disorder*” OR “language impairment*” OR “specific language impairment” OR SLI OR “communication disorder*” OR “speech disorder*” OR “speech sound disorder*” OR stutter* OR stammer* OR “fluency disorder*” OR “social communication disorder” OR “pragmatic language impairment” OR “social pragmatic communication” OR “oral language difficult*” OR “history of language disorder”) AND (anxiety OR depression OR “social anxiety” OR “psychological distress” OR well-being OR “well-being” OR loneliness OR “self-esteem” OR “social withdrawal” OR “mental health” OR “mental disorder*” OR “academic performance” OR GPA OR grade* OR retention OR attrition OR dropout OR persistence OR “academic engagement” OR participation OR “social integration” OR belonging OR “peer relation*” OR friendship* OR disclosure OR accommodation* OR “help-seeking”))	English; peer-reviewed journal articles; 2000 onward
CINAHL	Title/abstract/subject-	((“college student*” OR “university student*” OR undergraduate*	English; peer-

	term search	OR postgraduate* OR “graduate student*” OR “higher education” OR “tertiary education” OR universit* OR college*) AND (“developmental language disorder” OR DLD OR “language disorder*” OR “language impairment*” OR “specific language impairment” OR SLI OR “communication disorder*” OR “speech disorder*” OR “speech sound disorder*” OR stutter* OR stammer* OR “fluency disorder*” OR “social communication disorder” OR “pragmatic language impairment” OR “social pragmatic communication” OR “oral language difficult*” OR “history of language disorder”) AND (anxiety OR depression OR “social anxiety” OR “psychological distress” OR well-being OR “well-being” OR loneliness OR “self-esteem” OR “social withdrawal” OR “mental health” OR “mental disorder*” OR “academic performance” OR GPA OR grade* OR retention OR attrition OR dropout OR persistence OR “academic engagement” OR participation OR “social integration” OR belonging OR “peer relation*” OR friendship* OR disclosure OR accommodation* OR “help-seeking”))	reviewed journal articles; 2000 onward
Scopus	Title, abstract, keyword	TITLE-ABS-KEY((“college student*” OR “university student*” OR undergraduate* OR postgraduate* OR “graduate student*” OR “higher education” OR “tertiary education” OR universit* OR college*) AND (“developmental language disorder” OR DLD OR “language disorder*” OR “language impairment*” OR “specific language impairment” OR SLI OR “communication disorder*” OR “speech disorder*” OR “speech sound disorder*” OR stutter* OR stammer* OR “fluency disorder*” OR “social communication disorder” OR “pragmatic language impairment” OR “social pragmatic communication” OR “oral language difficult*” OR “history of language disorder”) AND (anxiety OR depression OR “social anxiety” OR “psychological distress” OR well-being OR “well-being” OR loneliness OR “self-esteem” OR “social withdrawal” OR “mental health” OR “mental disorder*” OR “academic performance” OR GPA OR grade* OR retention OR attrition OR dropout OR persistence OR “academic engagement” OR participation OR “social integration” OR belonging OR “peer relation*” OR friendship* OR disclosure OR accommodation* OR “help-seeking”))	English; journal articles; 2000 onward
Web of Science	Topic search	TS=((“college student*” OR “university student*” OR undergraduate* OR postgraduate* OR “graduate student*” OR “higher education” OR “tertiary education” OR universit* OR college*) AND (“developmental language disorder” OR DLD OR “language disorder*” OR “language impairment*” OR “specific language impairment” OR SLI OR “communication disorder*” OR “speech disorder*” OR “speech sound disorder*” OR stutter* OR stammer* OR “fluency disorder*” OR “social communication disorder” OR “pragmatic language impairment” OR “social pragmatic communication” OR “oral language difficult*” OR “history of language disorder”) AND (anxiety OR depression OR “social anxiety” OR “psychological distress” OR well-being OR “well-being” OR loneliness OR “self-esteem” OR “social withdrawal” OR “mental health” OR “mental disorder*” OR	English; articles; 2000 onward

	"academic performance" OR GPA OR grade* OR retention OR attrition OR dropout OR persistence OR "academic engagement" OR participation OR "social integration" OR belonging OR "peer relation*" OR friendship* OR disclosure OR accommodation* OR "help-seeking"))
