# Supplementary material for: Hidden Communication Needs in Higher Education: A Scoping Review of Developmental Communication Disorders, Mental Health, and Academic Participation
Source: Healthcare (Basel). 2026 Jun 21;14(12):1790. doi: 10.3390/healthcare14121790 (PMC13299988; doi:10.3390/healthcare14121790)
Supplement: Supplementary file 1 [file healthcare-14-01790-s001.zip › Supplementary Table S2 Full study characteristics of the included studies-updated.pdf]

Supplementary Table S2. Full study characteristics of the included studies

Study	Country/setting	Design	Participants/sample	Communication or communication-related profile	Data collection and analysis	Outcomes/review domains	Key findings relevant to this review
Azios et al. (2022) [8]	United States; university settings across multiple regions	Qualitative interpretative phenomenological analysis	7 women who stutter, aged 19-24, who were current or recent university students	Developmental stuttering; gendered lived experiences of women who stutter in university	In-depth semi-structured interviews and follow-up email questions; interpretative phenomenological analysis with member checking, audit trail, and bracketing	MH; AP; SP; IS	Women who stutter experienced university participation through the combined effects of stuttering, gender stereotypes, peer and family responses, disability services, therapy experiences, and interactions with authoritative figures. Supportive relationships, client-centred therapy, disclosure, and risk-taking supported confidence and participation, while discrimination, point deductions, negative staff responses, and stereotypes restricted participation.
He et al. (2025) [9]	China and Japan; higher education institutions in China and Japan	Qualitative interpretative phenomenological approach	22 undergraduate students who stutter; 11 Chinese and 11 Japanese	Developmental stuttering in Chinese and Japanese university students	Semi-structured interviews of 45-90 minutes via Zoom or Tencent Meetings, informed by the ICF model; reflexive thematic analysis using	MH; AP; SP; CT; IS	Students in both countries experienced communication barriers and negative psychological responses. Chinese students

					NVivo 14 with independent coding and triangulation		more often discussed professional counselling, while Japanese students emphasised public awareness. Cultural norms, including "face" in China and "reading the air" in Japan, shaped students' experiences and coping. Both groups reported insufficient institutional support.
Isaacs (2020) [10]	South Africa; two universities across honours and postgraduate programmes	Analytical autoethnography	Author as a person who stutters, with accounts involving university staff, lecturers, librarians, and selection panel members	Developmental stuttering and lived experience of stuttering in higher education	Retrospective recollection, lived experience, critical incidents, vignettes, and reflective journaling; analytical autoethnography using Felt's chronopolitics concept	MH; AP; SP; IS; CT	University time structures privileged speed, fluency, and immediate responsiveness, creating barriers for a student who stutters. The account described explicit exclusion from postgraduate pathways, dismissive institutional responses, and the absence of accommodation, arguing that fluency is not a necessary condition for academic competence.
Sasso et al. (2024) [11]	United States; predominantly white institutions and co-curricular spaces	Qualitative narrative inquiry	10 undergraduate students who stutter	Developmental stuttering; co-curricular participation and interpersonal relationships	Semi-structured interviews of approximately two hours, a demographic questionnaire, and consent	MH; AP; SP; IS	Students who stutter experienced an "academic oratory tax" through limited co-curricular

					forms; narrative and thematic analysis, audit trail, and member checking		participation, grade penalties, lack of accommodations, invisibility, social isolation, intimacy struggles, and microaggressions. The findings showed that accommodation often fails to extend into informal learning and belonging spaces.
Werle & Byrd (2021) [12]	United States; retrospective undergraduate experiences	Mixed methods survey	246 adults; 123 who stutter and 123 who do not stutter, matched for age; all had completed college courses requiring oral presentations	Developmental stuttering and professor perceptions during undergraduate oral presentations	Qualtrics survey using 16 visual analogue personality-trait ratings, comfort questions, forced-choice evaluation perception items, and open-ended responses; Wilcoxon signed-rank tests, logistic regression, chi-square, effect sizes, and thematic coding	MH; AP; SP; IS	Adults who stutter reported more negative professor perceptions on several traits and were less comfortable approaching professors than adults who did not stutter. Perceived self-assuredness predicted comfort approaching professors for feedback. Most adults who stutter identified stuttering as a primary factor shaping their oral presentation experiences.
Werle & Byrd (2022a) [13]	United States; online higher education instructor sample	Quantitative experimental 2 × 2 factorial study	158 college instructors who required oral presentations, from 34 states and 29 departments	Professor perceptions and evaluations of students who do and do not stutter; communication	Participants viewed one randomised video condition varying stuttering presence and communication competence, then completed	AP; SP; IS	High communication competence improved ratings regardless of stuttering. In low-competence conditions, the student who stuttered was

				competence manipulation	personality ratings and a modified oral presentation rubric; two-way ANOVAs and Bonferroni-Holm pairwise comparisons		rated less negatively and scored higher than the fluent speaker. In high-competence conditions, the student who stuttered was still rated as more nervous, shy, and self-conscious.
Werle & Byrd (2022b) [14]	United States; online higher education instructor sample	Quantitative experimental 2 × 3 factorial study	238 college instructors who required oral presentations, from 38 states and 37 departments	Self-disclosure of stuttering and communication competence in oral presentation evaluation	Participants viewed one of six randomised video conditions varying fluency, stuttering disclosure, and communication competence, then completed personality ratings and a modified oral presentation rubric; 2x3 ANOVAs, Bonferroni-Holm comparisons, and outlier removal using Tukey IQR	AP; SP; IS	Self-disclosure improved positive ratings regardless of communication competence. In low-competence conditions, the disclosure video received higher total scores than fluent or stuttering-only videos. High communication competence reduced evaluation differences, while disclosure eliminated negative perceptual differences for stereotypical traits.
Del Tufo & Earle (2020) [15]	United States; 4-year public university	Quantitative cross-sectional secondary data analysis	352 college students aged 18-35; classified as DLD, developmental dyslexia, typical development, or unclassified because of missing data; reported groups included 50 with DLD, 40 with developmental	History of developmental language disorder and/or developmental dyslexia	Standardised measures of reading, spoken language, nonverbal cognition, verbal working memory, phonological processing, rapid naming, and self-reported childhood diagnostic	AP; CT	Students with a history of DLD showed weaknesses in verbal working memory, nonword repetition, and reading fluency. Students with developmental dyslexia showed weaknesses in phonological

			dyslexia, and 132 typically developing students		history; structural equation modelling and confirmatory factor analysis		processing, nonword repetition, and rapid automatised naming. The findings suggest that different language and reading histories require differentiated academic supports in higher education.
Downey et al. (2000) [16]	United States; University of Colorado at Boulder	Quantitative comparative study across two studies	Study 1: 26 students in modified foreign-language classes and 27 in regular foreign-language classes. Study 2: 19 students in modified Latin grammar and 15 in regular Latin grammar	Dyslexia and persistent phonological processing difficulties affecting foreign-language learning	MLAT, WRAT-3, Nelson-Denny, Pig Latin, word/nonword repetition, grades, and Latin proficiency test; one-way ANOVA	AP; IS	Dyslexic college students showed lower foreign-language aptitude, phonological processing, reading, and spelling performance. However, students in modified Latin classes achieved comparable grades and proficiency to peers in regular classes, suggesting that slower-paced, structured, supported instruction can improve access to foreign-language learning.
Heiman & Precel (2003) [17]	Israel; Open University of Israel, distance education context	Quantitative comparative survey with open-ended items	191 college students with learning disabilities and 190 students without learning disabilities; aged 16-59	Learning disabilities with academic, language-related, and coping implications	Demographic questions, Learning Strategies Questionnaire, and Factors in Academic Success items; qualitative coding,	MH; AP; IS	Students with learning disabilities reported greater difficulties in humanities, social sciences, and foreign-language courses, greater exam stress and

					frequencies, chi-square tests, and ANOVA		concentration problems, and a stronger preference for special test conditions. They used more oral, visual, and unusual learning strategies, while students without learning disabilities used more written techniques. No GPA difference was reported.
Spigarelli et al. (2025) [18]	Canada; Université Laval, Québec	Quantitative single-group pre-post intervention	15 French Canadian university students with specific reading learning disorder or combined specific reading and spelling learning disorder; 87% comorbid; 6 with ADHD	Developmental dyslexia and dysgraphia / specific reading and spelling learning disorders	Three-week morphological and phonological awareness intervention; standardised pre- and post-tests of phonological awareness, morphological awareness, word/nonword reading, spelling, and reading comprehension; Wilcoxon signed-rank tests and rank biserial correlations	AP; IS	Students showed significant improvements in morphological awareness, nonword reading, irregular word spelling, text spelling, and reading comprehension after intervention. Changes in phonological awareness, regular word reading/spelling, and nonword spelling were not significant. The findings support the potential value of speech-language and literacy-informed intervention in higher education.
Wang et al. (2024) [19]	China; university context	Quantitative cross-sectional study with moderation analysis	223 second-year undergraduates; 100 with specific learning disabilities and 123 typically developing	Specific learning disabilities in Chinese undergraduates, with a focus on	Raven's Advanced Progressive Matrices, rapid naming task, backward digit span, State-Trait	MH; AP	In typically developing students, working memory, but not state anxiety, predicted academic

			students, matched on gender, grade, major, and university	anxiety, working memory, and academic performance	Anxiety Inventory state subscale, and first-year GPA standardised by the university; correlations, regression, PROCESS moderation, and Johnson-Neyman technique		performance. In students with specific learning disabilities, the interaction between state anxiety and working memory predicted academic performance. State anxiety negatively affected performance only for students with poor working memory. Processing speed was significant for both groups.
Icht et al. (2023) [20]	Israel; university context	Quantitative cross-sectional survey	1,582 undergraduates; gender-weighted sample of 50.2% female and 49.8% male; mean age 25.5 years	Self-identified cluttering characteristics	Validated self-report questionnaire including a lay definition of cluttering, self-identification, speech therapy history, and well-being measures; gender weighting, chi-square, ANOVA, MANOVA with faculty covariate, and transformation of BDI-II scores	MH; AP; SP	Twenty-three per cent of students self-identified as having cluttering characteristics. This group reported higher psychosomatic symptoms, depression, and stress, and lower self-esteem and happiness than students who did not self-identify with cluttering characteristics. Only a small proportion had received speech therapy.
Zukerman et al. (2024) [21]	Israel; Ariel University	Quantitative cross-sectional survey	1,201 Israeli undergraduates; 51.2% female; mean age 25.56; 276 self-identified with cluttering characteristics	Self-identified cluttering characteristics and self-inefficacy	Self-report questionnaire including lay definition of cluttering, self-identification, self-inefficacy scale, short BDI-II, HBSC	MH; SP	Self-identified cluttering characteristics predicted higher depression and psychosomatic symptoms, but not positive well-being. Self-

					psychosomatic symptoms, Subjective Happiness Scale, positive future orientation, and ADHD/autism diagnosis items; regression, moderation, chi-square, t-tests, and reliability analyses		inefficacy predicted all well-being outcomes and moderated associations between cluttering characteristics and depression and positive future orientation. ADHD diagnosis was unexpectedly associated with fewer depressive and psychosomatic symptoms.
Jansen et al. (2016) [22]	Belgium / Flanders; universities and university colleges	Quantitative survey/needs analysis	43 students with ASD, 43 matched typically developing students, and 30 student counsellors	ASD-related social communication and participation difficulties in higher education	Online questionnaire covering functioning problems, teaching/evaluation methods, and accommodation effectiveness; descriptive statistics, chi-square tests, and two-mode partitioning cluster analysis	AP; SP; IS	Students with ASD reported frequent problems with verbal and non-verbal communication, oversensitivity to change, and distinguishing main ideas from details. Problems occurred across common teaching and evaluation contexts. Extended exam duration, exam deferral, and smaller exam groups were perceived as helpful for some issues, but social communication difficulties could not be fully addressed by accommodations alone.
Plotts & Livermore (2007) [23]	United States; university neuropsychological	Single case study	One 20-year-old male college student with Russell-Silver	Russell-Silver syndrome with a nonverbal	Neuropsychological tests, parent/self-report, and	MH; AP; SP	The case showed superior verbal ability relative to weaker

	screening setting		syndrome	learning disability profile involving communication, academic, and psychosocial implications	school/medical history; qualitative case analysis and comparison to Rourke's nonverbal learning disability criteria		nonverbal, processing speed, visual memory, sensory-motor, mathematics, and writing fluency skills, alongside mild depression. The study identified a nonverbal learning disability profile in a student with Russell-Silver syndrome and highlighted the need for neuropsychological assessment and academic support.
Daniels et al. (2011) [24]	United States; two Midwestern universities	Mixed methods survey	328 university instructors from varied ranks and disciplines; 151 male and 168 female	Instructor perceptions of stuttering and students who stutter	12-item Likert questionnaire and open-ended question; descriptive statistics, one-way ANOVA with Bonferroni correction, effect sizes, and qualitative thematic coding	AP; SP; IS	Instructors held varied understandings of stuttering. Many were uncertain about its causes, and most opposed excusing students who stutter from oral tasks. Prior coursework in stuttering was associated with more positive attitudes. Qualitative responses showed willingness to support students but uncertainty about appropriate accommodations.
Meredith et al. (2012) [25]	Australia; public university websites and disability liaison services	Quantitative website audit/content analysis	39 Australian public university websites and disability liaison officers; no student participants	Institutional information and support visibility for students who stutter	Systematic review of disability webpages using 12 criteria and email enquiry to disability liaison officers;	IS; AP; CT	Only 13% of university websites provided information about alternative teaching or assessment arrangements for

					descriptive statistics and qualitative categorisation of responses		speech-impaired students, and no university met all 12 reviewed criteria. Approximately half of disability liaison officers responded to email enquiries. The study suggested that prospective or current students who stutter may struggle to make informed decisions about support.
Otrebski et al. (2024) [26]	Poland; university context	Quantitative quasi-experimental video vignette study	342 college students; 172 men and 170 women, aged 19-33; majors included administration, economics, paramedicine, and nursing	Attitudes toward a peer who stutters	Four-minute video of a conversation with a stuttering peer, followed by adapted ATDPS and PANAS-state measures; ANCOVA and Pearson correlations	SP; IS; CT	Negative feelings toward a peer who stutters were significantly associated with less positive attitudes, while positive feelings were not. Gender and academic major moderated relationships between feelings and attitudes. Attitudes toward a peer who stutters were less positive than toward peers with some other disabilities, suggesting ongoing stigma in higher education peer contexts.
Zong (2025) [27]	China; universities with compulsory public English courses involving	Qualitative interview study	12 Chinese non-English major postgraduates; 10 non-stuttering students and 2 students who stutter	English presentation anxiety, with a specific focus on students who stutter	One-to-one semi-structured interviews of 20-40 minutes, recorded and conducted in Chinese; thematic	MH; AP; CT	Non-stuttering students reported anxiety linked to low confidence, fear of negative evaluation, teacher/peer

	presentations				analysis informed by Young, Luo, and Kondo and Ying-Ling		interaction, vocabulary, Confucian culture, presentation difficulty, and grading. Students who stutter reported additional anxiety related to pronunciation disorders and fear of negative evaluation, with anxiety worsening stuttering. Coping involved practice, positive thinking, peer support, and efforts to avoid stuttering.
Ahmed (2025) [32]	Bangladesh; tertiary institutions in urban and semi-urban areas	Qualitative case study	15 tertiary students aged 21-25; 8 male, 7 female; undergraduate and graduate students from Business, Agriculture, Engineering, and Computer Science	Presentation-related speech disruptions, including stuttering, voice tremors, hesitations, and memory lapses, are associated with classroom presentation anxiety	Semi-structured interviews, classroom observations, and document analysis; thematic analysis using NVivo v14, following Braun and Clarke's six steps	MH; AP; CT	Fear of judgment, public speaking anxiety, English-medium language barriers, and insufficient preparation contributed to speech disruptions and physiological anxiety symptoms during presentations. Students used rehearsal, peer feedback, relaxation, and technology as coping strategies. Supportive classroom environments reduced anxiety, while rigid grading and judgmental audiences intensified it.
