# Supplementary material for: Hidden Communication Needs in Higher Education: A Scoping Review of Developmental Communication Disorders, Mental Health, and Academic Participation
Source: Healthcare (Basel). 2026 Jun 21;14(12):1790. doi: 10.3390/healthcare14121790 (PMC13299988; doi:10.3390/healthcare14121790)
Supplement: Supplementary file 1 [file healthcare-14-01790-s001.zip › Supplementary Table S3 Full study-level appraisal details of the included studies-updated.pdf]

Supplementary Table S3. Full study-level appraisal details of the included studies

Author	Clear Aims	Qual.Method	Design	Recruitment	Data Collection	Reflexivity	Ethics	Rigour	Findings Clear	Value
Azios et al. (2022) [8]	Yes	Yes	Yes	Yes	Yes	Yes	Yes	Yes	Yes	High
He et al. (2025) [9]	Yes	Yes	Yes	Yes	Yes	No	Yes	Yes	Yes	High
Isaacs (2020) [10]	Yes	Yes	Yes	No	No	Yes	Yes	Partial	Yes	Medium
Sasso et al. (2024) [11]	Yes	Yes	Yes	Yes	Yes	Yes	Yes	Yes	Yes	High
Werle & Byrd (2021) [12]	Yes	Partial	Yes	Yes	Yes	No	Yes	Yes	Yes	High
Werle & Byrd (2022a) [13]	Yes	No	Yes	Yes	Yes	No	Yes	Yes	Yes	High
Werle & Byrd (2022b) [14]	Yes	No	Yes	Yes	Yes	No	Yes	Yes	Yes	High
Del Tufo & Earle (2020) [15]	Yes	No	Yes	Yes	Yes	No	Yes	Yes	Yes	High
Downey et al. (2000) [16]	Yes	No	Yes	Yes	Yes	No	Yes	Partial	Yes	High
Heiman & Precel (2003) [17]	Yes	Partial	Yes	Yes	Yes	No	Yes	Yes	Yes	High
Spigarelli et al. (2025) [18]	Yes	No	Partial (no control group)	Yes	Yes	No	Yes	Partial	Yes	High
Wang et al. (2024) [19]	Yes	No	Yes	Yes	Yes	No	Yes	Yes	Yes	High
Icht et al. (2023) [20]	Yes	No	Yes	Yes	Yes	No	Yes	Yes	Yes	High
Zukerman et al. (2024) [21]	Yes	No	Yes	Yes	Yes	No	Yes	Yes	Yes	High
Jansen et al. (2016) [22]	Yes	No	Yes	Yes	Yes	No	Yes	Yes	Yes	High
Plotts & Livermore (2007) [23]	Yes	No	Yes	No (The parents referred	Yes	No	Yes	Yes	Yes	High

				the participant for assessment)						
Daniels et al. (2011) [24]	Yes	Partial	Yes	Yes	Yes	No	Yes	Yes	Yes	High
Meredith et al. (2012) [25]	Yes	No	Yes	No	Yes	No	No (no human participants)	Partial	Yes	Medium
Otrebski et al. (2024) [26]	Yes	No	Yes	Yes	Yes	No	Yes	Yes	Yes	High
Zong (2025) [27]	Yes	Yes	Yes	Yes	Yes	No	Yes	Partial	Yes	High
Ahmed (2025) [32]	Yes	Yes	Yes	Yes	Yes	No	Yes	Yes	Yes	High
